# Supplementary material for: Content-rich biological network constructed by mining PubMed abstracts
Source: BMC Bioinformatics. 2004 Oct 8;5:147. doi: 10.1186/1471-2105-5-147 (PMC528731; doi:10.1186/1471-2105-5-147)
Supplement: Additional File 5 — The original Chilibot query results of the term "long-term potentiation (LTP)" and 22 other terms, limiting the latest references analyzed to the years 1990, 1995, 2000, and 2004. [file 1471-2105-5-147-S5.bz2 › chilibotAdditionalFile5/ltp2004/html/LTP_PKC.html]

 


 **LTP** and **PKC** 
  
Found 181 abstracts in PubMed,  **30 abstracts were retrieved and analyzed**.  


---

 Search Google  |
 PDF files only 
|  EDU domain only 

---

**Interactive relationship** (e.g. stimulation, inhibition, etc)

- In further support of this idea, we found that a persistent, superoxide dependent increase in autonomous  **PKC**  activity isolated via DEAE column chromatography also was associated with  **LTP** .  Ref: 11826097 J Neurosci, 2002
- Taken together, our findings indicate that X XO induced potentiation and  **LTP**  share similar cellular mechanisms, including superoxide dependent increases in autonomous  **PKC**  activity.  Ref: 11826097 J Neurosci, 2002
- These experiments reveal, unexpectedly, the existence of two parallel kinase pathways, one involving CaMKII and the other PKA and  **PKC** , either of which can fully support the induction of  **LTP** , at this stage of development.  Ref: 14529720 Neuropharmacology, 2003
- In wild type mice, the  **LTP**  by weak TBS was abolished by inhibiting group I mGluR or protein kinase C  **PKC**  but not by blocking muscarinic acetylcholine receptors.  Ref: 12351712 J Neurosci, 2002
- These results suggest that both tetanically induced and phorbol ester induced  **LTP** , in the rat, share similar mechanisms which involve, at least in part, activation of  **PKC**  dependent mechanisms to increase quantal release from sympathetic preganglionic axon terminals.  Ref: 12742189 Auton Neurosci, 2003
- These results suggest that activation of CaMKII, PKA and  **PKC**  may be crucial for the induction and the early phase but not for the late phase maintenance of the spinal  **LTP** .  Ref: 14586032 J NeurophysiolJ Neurophysiol, 2003
- We conclude that the preconditioning inhibition of  **LTP**  induction is a complex process which involves activation of NMDAR, group I and group II mGluR, and intracellular cascades activating  **PKC**  and p38 MAP kinase.  Ref: 14521989 Neuroscience, 2003
- Considerable evidences have shown that calcium calmodulin dependent protein kinase II CaMKII, protein kinase A PKA and protein kinase C  **PKC**  are important for  **LTP**  in hippocampus.  Ref: 14586032 J NeurophysiolJ Neurophysiol, 2003
- Instead,  **LTP**  leads to rapid surface expression of NMDARs in a  **PKC**  and Src family dependent manner.  Ref: 11740502 Nat Neurosci, 2002
- Pharmacological and electrophysiological studies have shown that several neuronal functions, including long term potentiation  [ **LTP** ]   **LTP**  and long term depression LTD, specifically require  **PKC**  gamma.  Ref: 12417016 J Biochem (Tokyo), 2002
- Protein kinase M zeta PKM zeta is a newly described form of  **PKC**  that is necessary and sufficient for the maintenance of hippocampal long term potentiation  [ **LTP** ]   **LTP**  and the persistence of memory in Drosophila.  Ref: 12857744 J Biol Chem, 2003
- **PKC**  gamma deficient mice i have modified long term potentiation  [ **LTP** ]   **LTP**  in hippocampus, ii exhibit mild deficits in spatial and contextual learning iii exhibit impaired motor coordination due to persistent multiple innervations of climbing fibers on Purkinje cells, iv show attenuation of opioid receptor activation, and v show decreased effects of ethanol on type A of gamma aminobutyric acid GABA receptor.  Ref: 12417016 J Biochem (Tokyo), 2002
- However, inhibition of CaMKII together with inhibition of either PKA or  **PKC**  fully blocked the induction of  **LTP** .  Ref: 14529720 Neuropharmacology, 2003
- The preconditioning inhibition of  **LTP**  induction was shown by the use of kinase inhibitors to involve activation of  **PKC**  and p38 MAP kinase, but not p42 MAP kinase or tyrosine kinase.  Ref: 14521989 Neuroscience, 2003

**Parallel relationship** (e.g. studied together, co-existance, homology, etc.)

- The Roles of CaMKII, PKA and  **PKC**  in the Induction and Maintenance of  **LTP**  of C Fiber Evoked Field Potentials in Rat Spinal Dorsal Horn.  Ref: 14586032 J NeurophysiolJ Neurophysiol, 2003
- These results indicate that the signaling from group I mGluR to  **PKC**  involving Galphaq Galpha11 does not constitute the main pathway for  **LTP** , but it secures  **LTP**  induction by lowering its threshold in the hippocampal area CA1.  Ref: 12351712 J Neurosci, 2002
- Compatible with this hypothesis, inherited AChE R overexpression in transgenic mice resulted in perikaryal clusters enriched with PKCbetaII, accompanied by  **PKC**  augmented  **LTP**  enhancement.  Ref: 14581933 Mol PsychiatryMol Psychiatry, 2002
- Previous studies have shown DBA 2 mice to exhibit poor spatial contextual learning, impaired hippocampal  **LTP** , and hippocampal mossy fiber hypoplasia, as well as reduced hippocampal  **PKC**  activity and expression relative to C57BL 6 mice.  Ref: 12675922 J Neurochem, 2003
- In Str slices treated with the  **PKC**  and or PKA, the same stimulation induced long term potentiation  [ **LTP** ]  of field potentials similar to those observed in slices obtained from MW rats.  Ref: 12012161 Exp Brain Res, 2002
- In present work the roles of these three protein kinases in the induction and maintenance of  **LTP**  of C fiber evoked field potentials were evaluated by application of specific inhibitors of CaMKII KN 93 and AIP, PKA Rp CPT cAMPS and  **PKC**  chelerythrine and Go 6983 at the recording segments before and after  **LTP**  induction in Urethane anesthetized Sprague Dawley rats.  Ref: 14586032 J NeurophysiolJ Neurophysiol, 2002
